# Is Phthisis Hereditary

**Published:** 1875-05

**Authors:** Wm. A. Bennett

**Affiliations:** Syracuse


ART II—*Is Phthisis Hereditary?* A paper read before the Medical Association of Central New York, by WM. A. BENNETT, M. D., of Syracuse. Published by request of the Association.

Mr. President and Gentlemen of the Association:

But few years have elapsed, since the profession were unanimous in the affirmation of this question. It has to them an established fact, the result of observation, the ripe experience of the past, the absolute demonstration of dissection. But lately, some have questioned its truth affirming that at the most, a mere aptitude to contract the disease from external influences is all that is ever handed down from consumptive parent to child. Now it might not be of much practical moment to distinguish between direct hereditary transmission, organic or otherwise, and a promeness, bias, or liability, so powerful that its subject would be about as liable to it as if it were hereditary; if the latter were the fact and if it did not so alter and belittle the real state of the case, as to induce thoughtlessness and consequent carelessness in the unfortunate descendants of the consumptive and also in us all who are all deeply interested in their welfare.

It seems to me as clear that certain descendants of Phthisical, Scrofulous and Syphilitic parents, when the whole system, blood and solids, is thus affected, derive these diseases from their ancestors, as that they are themselves thus derived. Why should not bad health as well as good be transmitted to children, weak as well as strong constitution, and if this is so which we all admit, why do we make any doubt of the particular kind of diseases thus handed down. Indeed does it not seem the natural inference that like should produce like in this case as in others. In regard to contagious diseases we know this rule obtains, that small pox gives small pox and measles produce measles—And we natural expect this in hereditary as in contagious maladies, and that scrofula, phthisis and syphilis shall descend to the offspring and not some other diseases.

We may here very properly notice that everything in the germ and its derivation favors the idea of heredity. It is a secretion from the blood of each parent, and thus their entire and exact product. It is blood of their blood and bone of their bone in embryo, possessing their bodily, dynamical, chemical, vital and mental forces and characteristics. Here body and mind, material element and immaterial force, good or evil disposition, strong or weak emotion, firm or infirm constitution, clear or cloudy intellect all mingle and find expression in after growth and developement. This germ is the common receptacle and centre of the parental condition at the time its dual parts were formed and elaborated, and is in just such state as we might reasonably have expected to enable it to receive and return such condition. Hence it will reflect the parents more or less accurately, body and mind; and hence the bad health and wrong mental and moral qualities of ancestors shall pollute and curse their unhappy descendants, while their good health and pure strong minds shall transmit these desirable qualities to their fortunate offspring.

But let us come to facts in regard to this question for fortunately there are facts which bear directly upon it. Many consumptive women become pregnant in the active and later stages of this disease, either from choice as hoping to thereby to arrest or impede its progress, or from necessity by unsought intercourse with their husbands. For my personal satisfaction I examined two dead fœtuses of a hereditarily consumptive mother, in the last stage of the disorder at both conceptions, the one about six months after becoming pregnant, the other at nearly full term. Both lungs of the last were full of rounded miliary granulations, or unquestioned tubercles. The upper half of the right lung in the first case had many of the same character, some of them quite large and a few semi transparent. In both these cases there could be no doubt of the nature of the disease since there was its undoubted product. Now what else beside Phthisis killed these blighted offspring of a diseased mother, consumptively diseased from her mother's womb? They had never lived a separate existence, never breathed bad air, or been subjected to outside depressing influences. And I should here state that the mother was herself a good liver who loved and had as much of plain nutritous well cooked food as was best for her health and comfort; I think her own life was prolonged several years by this judicious course. Can any known case of cause and effect be plainer than that these diseased beings derived their disorder from her and did not have a mere leaning or bias towards it.

One fact justly applied is better than a thousand Hypotheses. Any comments on those cases can only weaken and dilute the evidence to one absolute and irrefragible, and I leave them to stand in all their native strength and power.

Sir Charles Scudamore speaks of a four months old child whose lungs he examined after its death of Phthisis, with extreme emaciation the mother being in the last stage of tuberculosis. He says "I never witnessed so extensive and remarkable a display of tubercles both granular and of a larger size, the former semi transparent and the latter gray in color. The lungs on each side upper and lower lobes, the liver, spleen, peritoneum and mesentary were universally studded with tubercles." Can any proof be more positive and convincing than this, showing as it does not only the direct descent of the disease from the actively diseased mother, but also its general nature by the extent of its deposits evidently laid down from the blood which must have been full of tubercles, or their elements being in this case as in so many others, a river of death to those through whom it flowed. In the begetting was the germ consumptive and thus originally diseased was continually kept in the line of its origin by its after support, until from the consumptive general state at inception it went on to have extensive and large localizations of tubercles, those terrible effects of a terribe disease. It is true, the child had lived and breathed, and perhaps nursed for four months after birth, but shall we therefore infer that its death was caused by phthisis from outside causes, or rather that the dying mother both in its origin and after life gave it her disorder. The latter is, it seems to me, the natural conclusion, and when the whole case is considered, too plain for argument. Like the mother was the child and was so because she directly made it such. In the embryotic secretion from her blood in the elements of that blood afterward filling and permeating every part of its being was transmitted that awful malady, which was a part of itself and an important and radical portion during its little hour of life.

Dr. C. I. B. Williams, of England, is a well known authority in consumption. He has personally examined and prescribed for from 25 to 30 thousand cases of the disease, and has analyzed and published the results so far as known of one thousand cases. In acuteness of perception, soundness of judgment and strength, and justness of reasoning he has no superior. He lays down the doctrine that Phthisis is hereditary, without one if or but, and sustains it by an array of facts perfectly overwhelming. The physicians of the greatest consumptive Hospital in the world that at Brompton, England, also agree with him upon this point, so that authority has settled the question so far as it can settle anything. In this instance it seems to me its decisions are abundantly sustained by the facts of the case.

A second proof of the hereditary nature of consumption is found in those cases where a consumptive parent dies having had consumptive children and the survivor healthy from birth marries again and with one also healthy and they have healthy children. Though I have never seen but two or three undoubted instances of this kind yet, even these were of great weight, since such marriages are infrequent and one positive proof is worth a host of negatives.

But thirdly, there is to me another and stronger evidence than the last, because I have seen so much more of it, to-wit: the successive deaths of whole families of children of consumptive parentage. I adduce a single example of this kind, similar to several others, occurring in an eastern state. The father was originally healthy and though he died of consumption at the age of sixty years, he was unwell only a few years before his decease. In fact

I thought he contracted the disorder from his family especially his wife, both of whose parents had died of this disease, and she herself was never free from it so far as I could learn; though she lived a few years after the birth of her youngest child, finally dying, aged about fifty, of consumption.

The children, six in number and all boys, deceased at ages varying from eighteen to about thirty, the last born dying youngest. All these unfortunates, except the very last, were born and lived for a number of years apparently in pretty fair health. But about the beginning of first manhood they began to droop and though some of them with great care and precaution lived a number of years, after this they all finally went the same way. This family was an intelligent and prudent one, of every external advantage for long life and good health. Their situation was healthful and pleasant; they were much in the open air and of good living and habits. Everything external was favorable to health and longevity, but the one inherent thing overbalanced all else, and hurried them all to premature graves. The fact that they were born consumptive was the sole reason of their early deaths. Many thus born are much aided by treatment, many cases of the disease arising *de novo* are often comparatively cured, but if both parents are actively diseased or even one at the time of the begetting, the chances are greatly against the progeny.

The last proof of the direct transmissibility of phthisis which I shall adduce is the analogous one of its descent in this way among animals. Thus a phthisical ram gave it to fifteen or twenty of his descendants. A Scotch bull also thus affected his calves, and guinea pigs innoculated with tubercle, produced tuberculous progeny. It is true that this method of proof is not conclusive since animals probably have different diseases from us, and are perhaps differently affected by the same, yet on the whole this fact is an important one in this case, since they do have the same disease, which in its symptoms, and pathology especially, is about the same as with us, and most likely obeys the same law of propagation.

On the whole the reasons for believing phthisis hereditary seem to me to be greatly proponderant, and I might therefore stop here but as some objections have been urged by some eminent members of the profession we will pay our respects to these, and see if they are sufficiently strong to overthrow the commonly received opinion.

And just here let us admire the ingenuity of these objectors They are all of them forced to admit that somehow the children of consumptive parents are more prone to that affection than others not thus circumstanced. But they say that parents do not transmit its organic predisposition, but do hand down an organization more prone to it when acted on by external causes. Now in some of the cases we have examined there were no external causes, and in the others these were favorable to health and yet their subjects died of phthisis, showing that the sole cause of such demise came directly from the parents. But take their view; what makes those organizations more liable to this affection than others, why do they upon the application of the same outside causes, which produce other diseases in others, have consumption? A bias indeed; a proneness certainly. But is it, or can it be anything else except the parental disease, inherent in the germ which after being latent for some time springs up under the influence of unhealthy exciting causes, nay sometimes against favorable circumstances?

But they tell us that all who have phthisis do not derive it from their parents and that therefore none of them do. Really this is a logical inference. Again they tell us a child may be so original as not to resemble either of his parents, true, but almost all children do and many of them very closely. A single and singular exception no more destroys a rule in this than in other cases. We cannot say because some children differ from their parents that such is the common fact since observation and reason at once prove the contrary. Is every act of coition successful even and yet this is the only way of successful propagation of the race. Do all syphilitics, after the primary stage, and when it is in the blood, transmit their terrible state to all their offspring, and yet do they not sometimes do this. And if, when such is the fact, you say these offspring have syphilis derived from their ancestors, I admit it and claim that true phthisis is often given in the same way. I have no statistics upon this common point in these two diseases, but I venture the assertion that actively diseased consumptive parents as often impart their disease, as any form of syphilis after the primary imparts itself. I have had demonstrative evidence that scarlatina is both, contagious and self-originating in several most convincing instances. Does the fact that it arises of itself deny the equally patent one of its contagion; do not different roads lead to the same place, different causes produce the same results? Because phthisis is self-originated more often is it not therefore transmitted at all? If everything is not accomplished by a powerful agent does it not therefore do anything. Such assumptions would set at naught all causes and effects and change universal odor into universal chaos.

But why is it necessary that disease if it descends at all shall go organically? If it is the same in the very young child or fœtus as in the parent it certainly goes into the germ by direct paternal action. Is phthisis organic at all? If anybody will tell what it is he will earn and receive the gratitude of his kind. I know not, and for the present purpose I care not, what it is. But it is the same in the child as in the parent. Are the life and instinct of the child organic and do they not descend from ancestors? Is not the young mentality conferred at the same time the bodily germ is created? And may not disease be like those transmitted to offspring even if it be not organically derived? It is not an organ, certainly though it is in them, as well as the life and the mind. There are a good many things intangible and yet we feel their effects. Many things are mysterious in their mode of action and in themselves. What do we know of immateriality or its union with matter, but that there is such a union and relation as best serves the ends of both we make no question. When we have proved that phthisis is organic, then we shall be called to admit its organic descent and not before. I do not say that it is organic, or not organic, but that as we have no proof of the former we have no right to assume that it must descend in that way. Whether it goes in the germ or blood or milk of ancestors, it is hereditary all the same, directly derived from them and I know of no reason why it should necessarily be organic in either case, though to be sure the two latter modes of transmission if they were admitted, might form that idea. But strength and force are carried along even by these and so may disease itself be. At any rate the disorder comes directly to the child from the parent in either instance, would not come if the parent had it not, and is the same in both.

But a good many children of consumptive parents or rather of one consumptive parent, for I have never known a healthy child where both were actively diseased, do not have Phthisis at all. Perhaps seventy-five per cent. of such children are free from its influence. And why, if the disorder is not generated or continued in these, is it generated and continued in any? Simply because facts with their natural inferences prove the reverse. Do twenty-five per cent. of all the children not born of phthisical ancestors have the disorder? Not by any means, but only about six per cent. or one-fourth of this proportion. This is of itself an argument in favor of its hereditary transmission and to be set aside needs a very strong array indeed of counter facts. But the question seems why do not all have it, and we reply this may be the correct answer: It is an admitted fact that one parent commonly influences the child more than the other, and that there is often a great preponderance in this respect. The difference in the power of giving anything else probably extends to the propagation of disease in the common germ. If the consumptive influence is weak in the one parent and the healthy one strong in the other, and especially if the healthy one be that of the mother, what prevents the stronger from overcoming the weaker in this case as in others and thus the germ itself be left healthy. Bad causes outside operate unfavorably, good ones favorably. One or the other predominating, and this rule, it seems to me, is especially operative here, when the condition of the whole germ and of all its parts would appear particularly favorable to such result. Hence the slightly consumptive germ of the one parent may be permeated with the strongly healthy one of the other, and thus finally become healthy, or its diseased state be completely overcome, even as weak protoplasm is rendered strong and available for healthy growth by proper and strengthening influences, or as any poison is neutralized and made harmless by the proper antidotes.

Let us now glance at what has been said. Phthisis is hereditary because it kills the fœtus in utero, and the young child in the arms of its mother when both or even one of the parents are consumptive at the time of their begetting. Because when a consumptive parent married to a healthy one, and having had consumptive children, dies, and the surviver marries again, this time with a healthy person, the offspring of this second union are healthy. Because whole and parts of families with phthisical parents, one or both die and commonly early of the same disorder. Because one-fourth of the children of consumptive progenitors die of phthisis. The objection that people contract it *de novo* and therefore none have it hereditarily is absurd.

When it can be shown that any and every known hereditary or even contagious disease *must always* be given to *every one* exposed to its influence it will be time to admit that phthisis must always be hereditarily transmitted. Nor is it necessary that the derivation from ancestors should be organic, but only that the same disorder is derived. Bodily elements and organs are necessary to our being but so enforces and life-immateriality as well as materiality is a part of us, and is as surely transmitted by the parent as the bodily elements. Let it first be shown that phthisis is itself organic before it is assumed that it must organically descend. If we have proved that whatever it is, it is hereditorily transmitted, our object is accomplished.

